# Prevalence of Angina Pectoris: An Analysis of the National Health Interview Survey (NHIS) Database

**DOI:** 10.7759/cureus.83076

**Published:** 2025-04-27

**Authors:** Awanwosa V Agho, Fatimot Disu, Alexander S Figueroa, Bernard Wiredu, Efeturi M Okorigba, Michael Olanite, Okelue E Okobi, Tazeen Noman

**Affiliations:** 1 Internal Medicine, Mercy Catholic Medical Center, Darby, USA; 2 General Internal Medicine, Salisbury NHS Foundation Trust, Salisbury, GBR; 3 Medicine, University of the East Ramon Magsaysay Memorial Medical Center (UERMMMC) College of Medicine, Quezon City, PHL; 4 Internal Medicine and Oncology, Saint James School of Medicine, Park Ridge, USA; 5 Internal Medicine, West Virginia University, Morgantown, USA; 6 Emergency Medicine, University Hospitals Dorset NHS Foundation Trust, Bournemouth, GBR; 7 Family Medicine, IMG Research Academy and Consulting LLC, Homestead, USA; 8 Family Medicine, Larkin Community Hospital Palm Springs Campus, Miami, USA; 9 Family Medicine, Lakeside Medical Center, Belle Glade, USA; 10 Internal Medicine, Dhaka Medical College, Dhaka University, Little Rock, USA

**Keywords:** adults, angina pectoris, nhis, prevalence, retrospective data analysis, trends

## Abstract

Background: Angina pectoris remains a significant public health concern, highlighting disparities in cardiovascular health influenced by demographic, socioeconomic, and geographic factors. Analyzing the prevalence of trends is crucial to addressing health inequities and informing targeted interventions. The study of National Health Interview Survey (NHIS) data from 2019 to 2023 allowed us to observe how the pandemic affected cardiovascular care utilization when it decreased in 2020 and later rebounded into 2023 while investigating shifts in reported angina prevalence rates among main groups. Angina pectoris condition-related research requires assessment of current trends for effective health inequities intervention and targeted intervention planning.

Objective: This study aims to examine the prevalence of angina pectoris among United States (US) adults from 2019 to 2023 and across demographic, socioeconomic, and geographic factors.

Method: Data from the NHIS were analyzed to determine the prevalence of angina pectoris, which was identified through self-reported diagnosis or symptoms. The identification of angina pectoris in the NHIS dataset was based on self-reported physician diagnosis alongside responses to definite survey questions regarding chest pain and discomfort consistent with the symptoms of angina. Angina pectoris was identified in the NHIS dataset based on self-reported physician diagnoses and responses to specific survey questions on chest pain or discomfort consistent with angina. Stratified analyses assessed variations in prevalence across key demographic, socioeconomic, and geographic factors over a five-year period. The statistical analyses included both inferential analyses and descriptive statistics, including hypothesis testing and confidence interval estimation, to evaluate associations and divergences within the data. The prevalence of angina was evaluated across socioeconomic, demographic, and geographic groups using stratified analyses.

Results: The overall prevalence of angina pectoris remained stable (1.5-1.7%) from 2019 to 2023. Higher rates were observed among males (1.8%), older adults (4.5% in those aged 75 years and older), and US-born individuals (1.6%). Disparities observed across race/ethnicity further revealed disparities, with American Indian/Alaska Native individuals (2.1%) and Black individuals (1.2%) showing distinct patterns. Geographic trends highlighted a higher prevalence in areas with high social vulnerability (1.7%). Socioeconomic disparities were notable, with lower-income individuals (<100% federal poverty level (FPL)) experiencing higher prevalence (2.8-3.1%) and elevated rates among those with lower educational attainment. Employment status influenced prevalence, with unemployed individuals showing higher rates (3.4%).

Conclusion: The prevalence of angina pectoris reflects persistent disparities across demographic, socioeconomic, and geographic factors. The findings highlight the need for policies that enhance access to preventive cardiovascular care, early screening, and intervention, as well as address the social determinants of health, to minimize disparities in underserved populations.

## Introduction

Angina pectoris, a cardinal symptom of myocardial ischemia, remains a significant public health concern due to its association with coronary artery disease (CAD), one of the leading causes of morbidity and mortality worldwide. Angina pectoris is mainly characterized by chest discomfort and pain resulting from reduced blood flow to the heart [1,2]. It is a key clinical manifestation of CAD, one of the leading causes of morbidity and mortality worldwide. Angina significantly impacts patients’ quality of life, limiting physical activity and increasing the risk of acute coronary events [3]. The condition is classified as stable or unstable angina based on the frequency and triggers of symptoms [4,5]. To ensure an accurate analysis of the prevalence trends and related risk factors, this study has focused on unstable angina. Understanding the prevalence, disparities, and risk factors of angina pectoris is crucial for addressing health inequities, guiding targeted interventions, and enhancing early diagnosis, management, and prevention of cardiovascular complications [6]. Such factors also influence the burden of angina and underline the pertinence of this study in guiding future public health interventions and resource allotment. Additionally, angina pectoris is a key driver of healthcare utilization, resulting in frequent outpatient visits, hospital admissions, and diagnostic testing that jointly impose a significant economic burden on healthcare systems throughout the globe.

Epidemiological data underscore the burden of angina pectoris in various populations. According to the Centers for Disease Control and Prevention (CDC), approximately 11 million adults in the United States experience angina annually [7]. According to the CDC, approximately 3.4 million adults aged 40 years and older in the United States were estimated to have angina between 2009 and 2012 [8]. Angina pectoris affected 3.5% of the Swedish general population in a study, with a median age of 57.4 years and a slight female predominance (51.6%) [9]. In India, 4.69% of older males and 7.02% of older females reported symptom-based angina, with higher odds observed among individuals with hypertension, diabetes, high cholesterol, or a family history of heart disease compared to their healthier counterparts [6].

The pathophysiology of angina pectoris involves an imbalance between myocardial oxygen supply and demand, primarily due to reduced coronary blood flow. This imbalance is often caused by atherosclerosis, where lipid-laden plaques form within the coronary arteries, narrowing the lumen and restricting blood flow [1]. Stable angina is typically associated with fixed stenosis, where myocardial ischemia occurs during exertion or stress due to increased oxygen demand [2]. In contrast, unstable angina arises from acute plaque rupture, thrombus formation, and endothelial dysfunction, leading to unpredictable ischemic episodes even at rest [3]. Additionally, vasospasm and microvascular dysfunction can contribute to angina by reducing coronary perfusion independently of obstructive disease [10,11]. Systemic factors such as inflammation, oxidative stress, and comorbid conditions like hypertension and diabetes exacerbate these mechanisms. This complex interplay underscores the necessity for comprehensive strategies that address both the structural and functional components of myocardial ischemia in angina management [1-6,10,11]. It is noteworthy that diabetes and hypertension, whose prevalence varies across ethnic and racial groups, significantly contribute to myocardial ischemia development through mechanisms that include microvascular impairment and endothelial dysfunction. The outcomes are further exacerbated by the socioeconomic disparities that limit healthcare access and delay treatment.

The National Health Interview Survey (NHIS) is a long-standing, population-based survey conducted annually in the United States to assess the health status, healthcare access, and behaviors of the civilian, non-institutionalized population. It provides nationally representative data on various health conditions, including angina pectoris, by collecting self-reported information. NHIS serves as a critical resource for tracking disease trends and informing public health policies [12]. To do this, the NHIS database captures the main socioeconomic aspects that include education, income, insurance, employment, and access to healthcare, thereby serving as socioeconomic status proxies in epidemiological studies. Despite not offering a Social Vulnerability Index (SVI) score, it takes in data on social determinants, including location, race, and disability, which are associated with external SVI metrics. Such variables permit a comprehensive assessment of socioeconomic disparities in angina pectoris prevalence.

This study aims to examine the prevalence of angina pectoris among United States (US) adults using NHIS data from 2019 to 2023, analyzing trends across demographic, socioeconomic, and geographic factors. Specifically, the objectives include identifying demographic, geographic, and socioeconomic disparities; exploring potential factors influencing these trends; and providing actionable insights to inform healthcare strategies and interventions. Thus, the study will assess the associations between geographic, demographic, and socioeconomic factors and prevalence, acknowledging the cross-sectional attribute of the NHIS dataset limits causal inferences. By analyzing these data, we aim to contribute to a deeper understanding of angina's evolving epidemiological landscape and support evidence-based decision-making for CVD prevention and management. Regarding the temporal trends, the study will analyze the year-over-year variations in the prevalence of angina from 2019 to 2023 with the objective of assessing the changes and trends in disparities across the different population subgroups. The findings of this study will offer data-driven insights that will guide the targeted healthcare interventions. Through highlighting the high-risk populations alongside regional variations, this study seeks to support policymakers with the allocation of resources, refining screening strategies, and developing customized prevention programs for effective CVD management.

## Materials and methods

Data source and study design

For data sources and study design, this study utilized annual data from the NHIS (2019-2023), which collects self-reported health-related information through structured interviews. The study has also focused on variables related to angina pectoris, demographics, and associated risk factors. Additionally, the cross-sectional design limited the study to identifying associations without evaluating causality and determining the direction of the relationship, as the data were collected at a single time point. To minimize potential confounding, respondents with a history of coronary intervention were excluded, in addition to those with incomplete data.

Study participants and questionnaires

The study included adults aged 18 years and older responding to NHIS standardized questionnaires that included items on angina pectoris (self-reported diagnosis or symptoms), comorbidities, lifestyle factors, and socioeconomic status. Specific questions from the NHIS Cardiovascular Health module were used to identify individuals with angina pectoris. Individuals with incomplete data for key variables were excluded from the analysis. Notably, self-reported angina might be limited by symptom misinterpretation and recall bias, as well as a lack of clinical verification that might affect accuracy. Nevertheless, subpopulation analyses by income and ethnicity/race were planned a priori to assess the disparities in cardiovascular health.

Data collection and quality assurance

NHIS data were collected through in-person interviews conducted by trained interviewers. Quality assurance measures included standardized interviewer training, validation of responses through follow-up questions, and the use of computer-assisted personal interviewing (CAPI) systems to minimize errors. NHIS data underwent rigorous quality control processes, including consistency checks and imputation for missing data. In the NHIS dataset, missing values were addressed through hot-deck imputation, which replaces missing responses with values provided by similar respondents. Additionally, for variables not imputed in the NHIS data, we employed listwise deletion, and with less than 5% missing data, the robustness of the results was confirmed through sensitivity analyses.

Variables of interest

For this study, the identification of angina pectoris was done using responses to NHIS survey items, including (a) a physician diagnosis question, "Have you ever been diagnosed with angina by a doctor or health professional?" and (b) symptom-based responses that are consistent with angina, including reports of chest pains and discomfort during physical activity. The International Classification of Diseases (ICD) codes were not employed, as NHIS does not allot ICD codes in publicly accessible datasets. As such, the classification was done based on standardized survey items designed to reflect clinically relevant diagnoses.

Additionally, the study's primary outcome variable was the prevalence of angina pectoris. Consequently, the key demographic variables included age (categorized into four groups, such as 18-44, 45-64, 65-74, and 75 years and older), gender, and race/ethnicity (American Indian or Alaska Native individuals only, Asian individuals only, Black individuals only, White individuals only, Native Hawaiian or other Pacific Islander individuals only, American Indian or Alaska Native and White individuals, or Black and White individuals), nativity (US-born/foreign-born), and geographic variables included the CDC Social Vulnerability Status Index (little to no, low, medium, or high social vulnerability). In contrast, socioeconomic factors such as educational level (less than a high school diploma, high school diploma, some college, college degree or higher), family income (less than 100% federal poverty level (FPL), 110% to less than 200% FPL, 200% and greater FPL), and employment status were also analyzed. Health-related variables included comorbidities (e.g., hypertension, diabetes), smoking status, and levels of physical activity (sedentary (low physical activity), light physical activity, moderate physical activity, vigorous physical activity, exercise tolerance/capacity, and cardiac rehabilitation physical activity). Notably, age was grouped into four key categories to align with the risk stages of cardiovascular and other national studies. Also, the income categorization was based on the FPL proportions, adhering to the standards of health disparity research. These groupings have been selected to enhance interpretability, statistical power, and consistency with the existing literature. Furthermore, the variables were selected to investigate their potential association with the presence of angina and to explore any disparities among population subgroups.

Data analysis and statistical methods

Descriptive statistics were used to calculate the prevalence of angina pectoris for each year. Trends over time were assessed using age-adjusted prevalence rates. Data were weighted using the NHIS sampling weights to ensure national representativeness. Age-adjusted prevalence rates and confidence intervals (CIs) were calculated to assess precision. Statistical significance was evaluated using the chi-square test and two-way analysis of variance (ANOVA), with a significance level of 0.05. All analyses were conducted using IBM SPSS Statistics for Windows, Version 30 (Released 2024; IBM Corp., Armonk, New York, United States). Calculation of age-adjusted prevalence rates was performed using direct standardization of the 2000 United States Census population to ascertain comparability across the study years and to account for the demographic shifts. A chi-square test was employed to effectively compare the prevalence rates across years, while a two-way ANOVA was used to assess associations between categorical variables and prevalence trends, without inferring causal relationships. Although logistic regression was considered, it was not used, as the focus was primarily on general trends rather than individual risk factors. Future studies might explore multivariable models.

Furthermore, this study employed ANOVA and regression techniques to conduct statistical analyses. Thus, the calculated F-statistic (critical F-value) was derived to evaluate the variance comparisons and model fit, enabling the determination of statistically significant differences between the groups. Consequently, the critical F-value (F-crit), derived from the F-distribution table at the specified significance level (α) and degrees of freedom, played the crucial role of a threshold for rejecting the null hypothesis. Additionally, the critical t-value (t-crit) was appropriately referenced from the t-distribution table, based on the selected α level and degrees of freedom, to assess the significance of the t-tests. To address potential clustering effects arising from the NHIS's complex survey design, Taylor series linearization was employed to adjust the standard errors accordingly.

Ethical considerations

The study utilized publicly available, de-identified NHIS data, ensuring confidentiality and compliance with ethical standards. As the data are secondary and anonymized, Institutional Review Board (IRB) approval was not required. Ethical guidelines from the NHIS and the Declaration of Helsinki were strictly followed.

## Results

The overall prevalence of angina pectoris among adults in the US showed slight fluctuations between 2019 and 2023. In 2019, the prevalence was 1.7 (95% CI: 1.3-2.1), which decreased to 1.5 (95% CI: 1.2-1.8) in 2020 and remained the same in 2021. It slightly increased to 1.6 (95% CI: 1.3-1.9) in 2022 and remained stable at 1.6 (95% CI: 1.2-2.0) in 2023. The age-adjusted prevalence of diagnosed angina pectoris significantly differed for all variables over the years, with a p-value of <0.05.

Based on gender

Gender-specific trends revealed that females consistently reported lower prevalence rates compared to males. For females, the prevalence declined from 1.6 (95% CI: 1.1-2.1) in 2019 to 1.3 (95% CI: 1.0-1.7) in 2020 and 2021 before slightly rising to 1.4 (95% CI: 1.0-1.8) in 2022 and 2023. Males demonstrated higher and more stable rates, with a prevalence of 1.7 (95% CI: 1.2-2.2) in 2019, peaking at 1.9 (95% CI: 1.4-2.4) in 2022, and slightly decreasing to 1.8 (95% CI: 1.3-2.3) in 2023. The statistical significance of year-to-year changes was assessed through the performance of trend analyses and the evaluation of CI overlaps. Table 1 presents the annual prevalence of angina pectoris, along with the corresponding CIs, across various demographic characteristics such as gender, race/ethnicity, age groups, and nativity for the years 2019 to 2023. The reduced prevalence rates in females are attributable to gender differences in CVD presentations and healthcare-seeking attitudes, even as women experience atypical symptoms that result in underdiagnosis, and males are increasingly liable to report their symptoms and consequently seek medical attention. In males, the stable prevalence can be attributed to lifestyle and biological factors that include a higher CVD risk factor burden, alongside the divergences in occupational exposures and levels of physical activity.

**Table 1:** Assessing the prevalence of angina pectoris trends by demographic characteristics using t-test and ANOVA - indicates not available CI: confidence interval

Characteristics	2019	2020	2021	2022	2023	Statistical test value	p-value
Total 18 years or over, age-adjusted	1.7 (1.5-1.9)	1.5 (1.3-1.6)	1.5 (1.4-1.7)	1.6 (1.5-1.8)	1.6 (1.4-1.8)	-	-
Prevalence of angina pectoris (95% CI) based on gender							
Female	1.6 (1.4-1.9)	1.3 (1.1-1.5)	1.3 (1.1-1.5)	1.4 (1.2-1.6)	1.4 (1.2-1.6)	t critical: 2.30	<0.05
Male	1.7 (1.5-2.0)	1.6 (1.4-1.9)	1.7 (1.5-2.0)	1.9 (1.7-2.2)	1.8 (1.6-2.1)		
Prevalence of angina pectoris (95% CI) based on race/ethnicity							
American Indian or Alaska Native only	1.7 (0.5-4.1)	-	2.8 (1.1-5.8)	-	2.1 (0.7-4.7)	F-value: 31.25, F crit value: 7.7	<0.05
Asian only	1.1 (0.6-1.7)	0.9 (0.4-1.6)	1.0 (0.6-1.6)	1.4 (0.8-2.2)	0.8 (0.5-1.4)		
Black only	1.4 (1.0-1.9)	1.4 (0.9-2.1)	1.0 (0.7-1.5)	1.1 (0.8-1.6)	1.2 (0.8-1.7)		
White only	1.8 (1.6-2.0)	1.6 (1.4-1.8)	1.6 (1.5-1.8)	1.8 (1.6-2.0)	1.8 (1.6-2.0)		
American Indian or Alaska Native and White	-	2.2 (0.7-5.1)	-	-	-		
Black and White	-	0.1 (0.0-3.2)	1.1 (0.1-4.4)	0.7 (0.0-4.5)	-		
Prevalence of angina pectoris (95% CI) based on age groups							
18-44 years	0.5 (0.3-0.6)	0.3 (0.2-0.5)	0.4 (0.3-0.5)	0.4 (0.2-0.5)	0.4 (0.3-0.6)	F-value: 159.4, F crit value: 3.49	<0.05
45-64 years	1.7 (1.4-2.0)	1.5 (1.2-1.7)	1.6 (1.4-2.0)	1.5 (1.2-1.8)	1.8 (1.5-2.2)		
65-74 years	3.4 (2.9-4.0)	3.1 (2.6-3.6)	2.9 (2.4-3.5)	4.3 (3.5-5.1)	3.2 (2.7-3.8)		
75 years and over	5.7 (4.8-6.7)	5.1 (4.4-6.0)	4.8 (4.0-5.7)	4.9 (4.1-5.8)	4.5 (3.8-5.3)		
Prevalence of angina pectoris (95% CI) based on nativity							
US-born	1.7 (1.5-1.9)	1.6 (1.4-1.8)	1.6 (1.4-1.8)	1.8 (1.6-2.0)	1.6 (1.4-1.8)	t critical: 2.30	0.01
Foreign-born	1.4 (1.0-1.9)	0.8 (0.5-1.0)	1.3 (1.0-1.7)	1.2 (0.9-1.6)	1.5 (1.1-2.0)		

Based on races

The prevalence of angina pectoris exhibited distinct patterns across racial and ethnic groups during the study period, with accompanying CIs highlighting variability (Table 1). Among American Indian or Alaska Native adults, prevalence fluctuated from 1.7 (95% CI: -1.9 to 5.3) in 2019 to a peak of 2.8 (95% CI: -1.9 to 7.5) in 2021, before declining to 2.1 (95% CI: -1.9 to 6.1) in 2023. For Asian adults, prevalence remained consistently low, ranging from 0.8 (95% CI: -0.1 to 1.7) in 2023 to a high of 1.4 (95% CI: -0.1 to 2.9) in 2022. Black adults exhibited a gradual increase from 1.0 (95% CI: 0.2-1.8) in 2021 to 1.2 (95% CI: 0.3-2.1) in 2023, following stable levels of 1.4 (95% CI: 0.2-2.6) in 2019 and 2020. Among White adults, the prevalence was relatively stable, varying narrowly between 1.6 (95% CI: 1.3-1.9) in 2021 and 1.8 (95% CI: 1.4-2.2) in other years. Data for Native Hawaiian or other Pacific Islander individuals was unavailable, while prevalence among multiracial groups such as American Indian and White individuals, and Black and White individuals, was inconsistently reported.

Based on age

**Figure 1:**
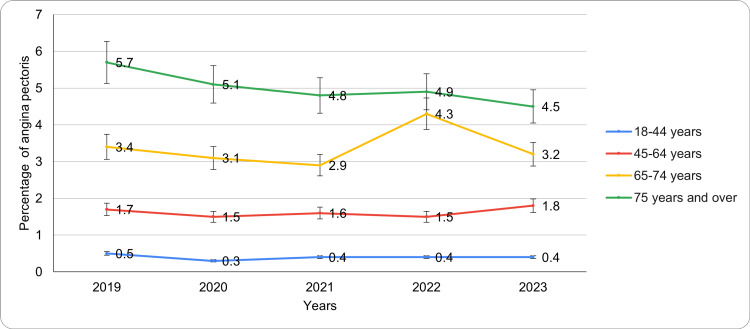
Angina pectoris trends based on age during study period

The prevalence of angina pectoris increased consistently with age across all years, reflecting an age-related trend (Figure 1). Among adults aged 18-44 years, prevalence remained low and stable, ranging from 0.3 (95% CI: 0.0-0.6) in 2020 to 0.5 (95% CI: 0.2-0.8) in 2019, with no significant changes observed through 2023. In the 45-64 years age group, prevalence exhibited slight fluctuations, starting at 1.7 (95% CI: 1.1-2.3) in 2019, decreasing to 1.5 (95% CI: 1.0-2.0) in 2020 and 2022, and rising to 1.8 (95% CI: 1.1-2.5) in 2023. Among adults aged 65-74 years, prevalence peaked in 2022 at 4.3 (95% CI: 2.7-5.9), with lower levels recorded in 2019 (3.4 (95% CI: 2.3-4.5)) and 2023 (3.2 (95% CI: 2.1-4.3)). Adults aged 75 years and older consistently demonstrated the highest prevalence, beginning at 5.7 (95% CI: 3.8-7.6) in 2019, with a gradual decline to 4.5 (95% CI: 3.0-6.0) in 2023. The observed rise in angina prevalence with age may reflect the cumulative cardiovascular risk exposure alongside the physiological changes that include reductions in myocardial perfusion and arterial stiffness. Further, the dissimilarities in symptom perception, access to healthcare, and healthcare-seeking attitudes might additionally influence angina diagnosis rates in different groups. For instance, in the oldest persons, variable diagnostic practices and survivor biases may be highly liable to contribute significantly to the consistently higher prevalence rates.

Based on nativity

The prevalence of angina pectoris displayed distinct trends based on nativity (Table 1). Among US-born individuals, prevalence remained relatively stable over the five years, ranging from 1.6 (95% CI: 1.2-2.0) in 2020, 2021, and 2023 to a slight increase of 1.8 (95% CI: 1.4-2.2) in 2022. This consistency may highlight a steady burden of angina pectoris within the US-born population. In contrast, the foreign-born population exhibited greater variability. The prevalence started at 1.4 (95% CI: 0.5-2.3) in 2019, then dropped significantly to 0.8 (95% CI: 0.3-1.3) in 2020. A gradual increase followed, reaching 1.3 (95% CI: 0.6-2.0) in 2021, 1.2 (95% CI: 0.5-1.9) in 2022, and peaking at 1.5 (95% CI: 0.6-2.4) in 2023. It is noteworthy that the diversity in the foreign-born populations, alongside factors such as the length of residency in the United States and language proficiency, may affect access to healthcare and the reporting of symptoms, possibly clarifying the observed variability in the prevalence of angina. Despite our dataset lacking these variables, we propose that they should be included in prospective studies to facilitate a better understanding of the trends.

Based on the CDC SVI

The prevalence of angina pectoris showed variation across levels of social vulnerability, as categorized by the CDC SVI. The variations in prevalence, based on the CDC SVI, are presented in Table 2 below.

**Table 2:** Assessing prevalence of angina pectoris trends by geographic characteristics and socioeconomic characteristics using t-test and ANOVA - indicates not available GED: General Educational Development; FPL: federal poverty level; CI: confidence interval; CDC: Centers for Disease Control and Prevention

Characteristics	2019	2020	2021	2022	2023	Statistical test value	p-value
Prevalence of angina pectoris (95% CI) based on CDC Social Vulnerability Index							
Little to no social vulnerability	1.3 (1.0-1.6)	1.4 (1.0-1.7)	1.3 (1.0-1.6)	1.7 (1.3-2.1)	1.3 (1.0-1.8)	F-value: 3.71, F crit value: 3.49	0.042
Low social vulnerability	1.5 (1.2-1.8)	1.3 (1.1-1.6)	1.3 (1.1-1.6)	1.7 (1.4-2.1)	1.5 (1.2-1.8)		
Medium social vulnerability	1.7 (1.4-2.1)	1.6 (1.3-1.9)	1.7 (1.5-2.1)	1.7 (1.4-2.0)	1.7 (1.4-2.1)		
High social vulnerability	2.1 (1.7-2.6)	1.5 (1.2-1.9)	1.6 (1.3-2.0)	1.5 (1.3-1.9)	1.7 (1.4-2.0)		
Prevalence of angina pectoris (95% CI) based on employment status							
Employed	0.7 (0.6-0.9)	0.6 (0.5-0.8)	0.6 (0.5-0.8)	0.6 (0.5-0.8)	0.8 (0.7-1.0)	F-value: 139.1, F crit value: 2.71	<0.05
Not employed	3.4 (3.0-3.8)	2.8 (2.5-3.2)	3.0 (2.7-3.3)	3.4 (3.0-3.9)	3.0 (2.6-3.4)		
Full-time	0.6 (0.5-0.8)	0.5 (0.4-0.7)	0.6 (0.4-0.7)	0.5 (0.4-0.6)	0.7 (0.6-0.9)		
Part-time	1.1 (0.8-1.6)	1.0 (0.6-1.5)	0.8 (0.6-1.2)	1.2 (0.8-1.8)	1.2 (0.8-1.6)		
Not currently employed but has worked previously	3.6 (3.2-4.0)	2.9 (2.6-3.3)	3.1 (2.8-3.5)	3.6 (3.2-4.1)	3.2 (2.8-3.6)		
Not currently employed and has never worked	1.3 (0.6-2.5)	-	1.4 (0.5-2.8)	0.8 (0.2-2.2)	0.6 (0.1-1.8)		
Prevalence of angina pectoris (95% CI) based on educational level							
Less than high school diploma	3.4 (2.6-4.3)	2.7 (1.9-3.6)	2.3 (1.7-3.0)	3.0 (2.2-3.9)	3.1 (2.3-4.0)	F-value: 51.4, F crit value: 3.49	<0.05
High school diploma or GED	2.0 (1.7-2.4)	1.9 (1.6-2.3)	1.9 (1.5-2.3)	2.2 (1.9-2.7)	2.1 (1.7-2.5)		
Some college	2.1 (1.8-2.5)	1.6 (1.3-1.9)	2.2 (1.8-2.6)	2.0 (1.7-2.4)	1.9 (1.5-2.3)		
College degree or higher	1.0 (0.8-1.2)	1.0 (0.8-1.2)	1.0 (0.8-1.2)	1.1 (0.9-1.3)	1.2 (1.0-1.4)		
Prevalence of angina pectoris (95% CI) based on family income							
Less than 100% FPL	3.0 (2.3-3.8)	2.6 (1.9-3.5)	2.4 (1.8-3.1)	3.1 (2.4-4.0)	2.8 (2.1-3.5)	F-value: 81.8, F crit value: 4.45	<0.05
100% to less than 200% FPL	2.1 (1.7-2.5)	2.1 (1.7-2.6)	2.3 (1.9-2.8)	2.1 (1.7-2.7)	2.0 (1.6-2.5)		
200% and greater FPL	1.4 (1.2-1.6)	1.1 (1.0-1.3)	1.2 (1.0-1.4)	1.3 (1.1-1.5)	1.3 (1.2-1.5)		

**Figure 2:**
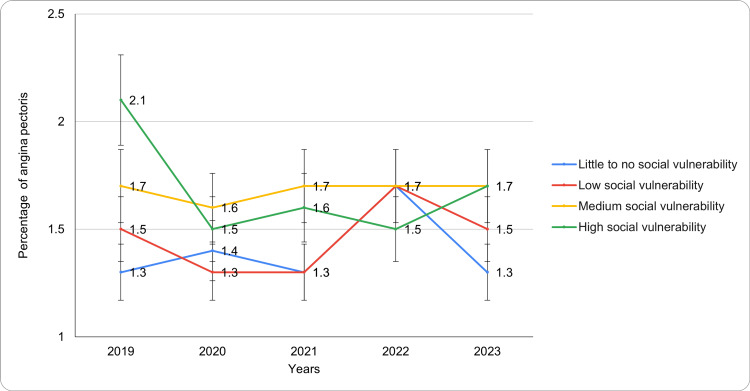
Angina pectoris trends based on the CDC Social Vulnerability Index during the study period CDC: Centers for Disease Control and Prevention

Among individuals with little to no social vulnerability, prevalence remained stable at 1.3% (95% CI: 0.7-1.9) in 2019 and 2021, rose to 1.4% (95% CI: 0.7-2.1) in 2020, peaked at 1.7% (95% CI: 0.9-2.5) in 2022, and returned to 1.3% (95% CI: 0.5-2.1) by 2023 (Figure 2). For those with low social vulnerability, prevalence decreased from 1.5% (95% CI: 0.9-2.1) in 2019 to 1.3% (95% CI: 0.6-2.0) in 2020 and 2021, increased to 1.7% (95% CI: 0.9-2.5) in 2022, and declined to 1.5% (95% CI: 0.8-2.2) in 2023. In the medium social vulnerability group, prevalence remained steady from 2019 to 2023, ranging from 1.6 (95% CI: 0.9-2.3) to 1.7 (95% CI: 0.9-2.5), with no notable peaks or declines. For the high social vulnerability group, prevalence started at 2.1 (95% CI: 1.1-3.1) in 2019, dropped to 1.5 (95% CI: 0.7-2.3) in 2020 and 2022, and slightly increased to 1.7 (95% CI: 0.8-2.6) by 2023. Numerous confounders, including healthcare access patterns and comorbidities, are likely to influence the correlations between social vulnerability and the prevalence of angina. Though this study did not adjust for such variables, it is recommended that prospective analyses should assess their potential mediating effects. Regardless of the modest differences, the observed higher burden of angina in vulnerable populations has significant public health and clinical implications, reflecting the systemic inequities in cardiovascular care. This study's findings support the requirement for increasingly nuanced social vulnerability stratification and alignment with extant literature, providing novel insights into the way such disparities evolved in the course of the COVID-19 pandemic.

Based on employment status

The prevalence of angina pectoris varied significantly across different employment statuses (Table 2). Among employed individuals, prevalence remained consistently low, ranging from 0.6 (95% CI: 0.4-0.8) in 2020-2022 to a slight increase of 0.8 (95% CI: 0.6-1.0) in 2023. Conversely, the not-employed group showed the highest prevalence, peaking at 3.4 (95% CI: 3.0-3.9) in 2019 and 2022, with slight declines to 2.8 (95% CI: 2.5-3.2) in 2020 and 3.0 (95% CI: 2.6-3.4) in 2023. For full-time workers, prevalence remained stable, fluctuating between 0.5 (95% CI: 0.4-0.7) and 0.6 (95% CI: 0.4-0.7), with a peak of 0.7 (95% CI: 0.6-0.9) in 2023. Part-time workers exhibited prevalence levels ranging from 1.0 (95% CI: 0.6-1.5) to 1.2 (95% CI: 0.8-1.6), with the lowest value of 0.8 (95% CI: 0.6-1.2) recorded in 2021. Among individuals not currently employed but with prior work experience, the prevalence ranged from 2.9 (95% CI: 2.6-3.3) in 2020 to 3.6 (95% CI: 3.2-4.1) in both 2019 and 2022. Those not currently employed and who had never worked experienced the highest prevalence in 2021, at 1.4 (95% CI: 0.5-2.8), with a notable decline to 0.6 (95% CI: 0.1-1.8) in 2023. The observed higher angina prevalence among non-employed persons can be attributed to factors that include chronic stress, fiscal insecurity, and limited healthcare access. Also, part-time employees have indicated increased angina prevalence, potentially reflecting underemployment, alongside pre-existing health conditions. Furthermore, confounding factors such as age, socioeconomic status, and comorbidities may influence the relationship, underscoring the need for appropriately adjusted analyses to isolate the effects of employment status on angina risk.

Based on educational level

**Figure 3:**
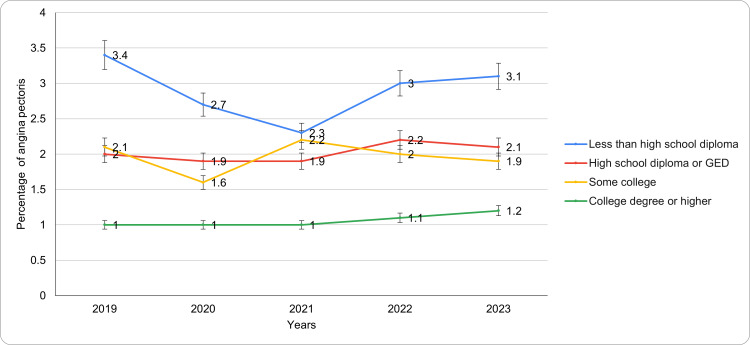
Angina pectoris trends based on educational level during the study period GED: General Educational Development

Educational attainment exhibited an inverse relationship with the prevalence of angina pectoris, with higher education levels associated with lower prevalence (Figure 3). Individuals with less than a high school diploma had the highest prevalence, starting at 3.4 (95% CI: 2.5-4.3) in 2019, declining to 2.3 (95% CI: 1.5-3.1) in 2021, and slightly rising to 3.1 (95% CI: 2.2-4.0) in 2023. Those with a high school diploma or GED demonstrated relatively stable prevalence, ranging from 1.9 (95% CI: 1.5-2.3) in 2020-2021 to 2.2 (95% CI: 1.7-2.7) in 2022. Among individuals with some college education, prevalence fluctuated, peaking at 2.2 (95% CI: 1.6-2.8) in 2021 and dropping to 1.6 (95% CI: 1.0-2.2) in 2020 and 1.9 (95% CI: 1.2-2.6) in 2023. Individuals with a college degree or higher exhibited the lowest and most stable prevalence, consistently at 1.0 (95% CI: 0.8-1.2) between 2019 and 2021, with slight increases to 1.1 (95% CI: 0.9-1.3) in 2022 and 1.2 (95% CI: 1.0-1.4) in 2023. Notably, the trend shows that educational attainment may have a significant impact on health literacy, cardiovascular risk factor awareness, and healthcare-seeking attitudes, influencing the recognition and reporting of angina. Thus, individuals with lower educational attainment may be likely to experience difficulties understanding angina symptoms and accessing care, which contributes to the observed disparities. In this regard, it is recommended that targeted and culturally appropriate health education and screening programs be primarily delivered through community-based contexts to enhance awareness while also encouraging early healthcare engagement among these populations.

Based on family income level

Family income levels were inversely correlated with the prevalence of angina pectoris (Table 2). Among individuals earning less than 100% of the FPL, prevalence declined from 3.0 (95% CI: 2.2-3.8) in 2019 to 2.4 (95% CI: 1.6-3.2) in 2021, peaking again at 3.1 (95% CI: 2.2-4.0) in 2022. For those earning between 100% and less than 200% of the FPL, prevalence remained relatively stable, ranging from 2.0 (95% CI: 1.5-2.5) in 2023 to 2.3 (95% CI: 1.8-2.8) in 2021. Individuals earning 200% or more of the FPL demonstrated the lowest prevalence, declining from 1.4 (95% CI: 1.0-1.8) in 2019 to 1.1 (95% CI: 0.8-1.4) in 2020 and stabilizing at 1.3 (95% CI: 1.0-1.6) from 2021 to 2023. The inverse correlations between angina prevalence and family income can be attributed to limited access to healthcare, lower levels of health literacy, and increased exposure to stress and cardiovascular risk factors among low-income persons. These trends are also likely to have been influenced by the COVID-19 pandemic through disproportionate impact on low-income populations through healthcare disruptions, job losses, and increased stress levels, which might clarify the fluctuations in angina prevalence rates, especially the dip observed in 2021 and the elevation that occurred in 2022 among individuals below 100% FPL. Consequently, in the 100% to <200% FPL group, the angina prevalence rate remained comparatively stable, which can be attributed to overlapping CIs as opposed to the statistically significant changes.

## Discussion

The findings of this study highlight several factors influencing the prevalence of angina pectoris among adults over a five-year period, providing insights into disparities across demographic, geographic, socioeconomic, and health-related characteristics. The overall prevalence of angina remained relatively stable, with minor fluctuations between 2019 and 2023, ranging from 1.5% to 1.7%. Despite this stability, significant differences were observed across various subgroups, indicating that sociodemographic, geographic, and economic disparities play a crucial role in the burden of angina [13].

Gender differences in angina prevalence were evident, with males consistently showing higher rates (1.6% to 1.9%) than females (1.3% to 1.4%) across all years. This aligns with existing literature, such as Zujie et al., which suggests that males are more prone to cardiovascular conditions, possibly due to hormonal, lifestyle, and occupational differences. However, the narrowing gender gap in recent years calls for further investigation into shifting behavioral or environmental factors [13-15]. The observed gender disparities in the prevalence of angina are often influenced by behavioral and biological factors. For instance, estrogen provides premenopausal females with certain levels of cardiovascular protection, possibly delaying the onset of symptoms [13,14]. On the contrary, men have higher smoking rates, reduced healthcare engagement, and increased levels of occupational stress, which not only increases their risk but also contributes to the observed differences [13,14].

Racial and ethnic disparities were also observed, with White adults consistently showing higher rates of angina compared to other groups [13]. Black adults exhibited a slightly lower prevalence, while Asian adults had the lowest rates, ranging from 0.8% to 1.4%. Data for other racial groups, such as Native Hawaiians, Pacific Islanders, and mixed-race individuals, were often unreliable or unavailable, highlighting the need for better representation and data collection among these populations. The study by Quashie et al. suggests that differences may influence these disparities in healthcare access, lifestyle factors, and socioeconomic conditions. The higher prevalence among White adults may reflect factors such as healthcare access, socioeconomic status, or genetic predispositions [13-17]. In comparison, the lower prevalence among Asian adults could be related to protective cultural or dietary factors [13-17].

Age-related trends revealed a marked increase in the prevalence of angina with advancing age. Adults aged 18-44 years consistently reported the lowest prevalence, around 0.3% to 0.5%, whereas those aged 75 years and older experienced the highest prevalence, ranging from 4.5% to 5.7%. The prevalence among individuals aged 65-74 years also remained substantial, highlighting the increasing cardiovascular risk with aging. These findings emphasize the critical importance of age-specific prevention and management strategies for cardiovascular diseases (CVDs), particularly among older adults who are at heightened risk due to age-related physiological changes and accumulated exposure to risk factors [13,14,17].

Nativity emerged as a key factor influencing angina prevalence, with US-born individuals consistently showing a slightly higher rate (1.6% to 1.8%) compared to foreign-born individuals (0.8% to 1.5%). This difference may stem from variations in healthcare access, cultural attitudes toward health, and lifestyle habits. A study by Lee et al. found that the prevalence of CVD was lower among Asian individuals than White individuals, regardless of nativity (OR ≥ 15 years = 0.5 (95% CI: 0.5-0.6), OR US-born = 0.7 (95% CI: 0.6-0.8)). Foreign-born individuals might benefit from healthier dietary and physical activity patterns from their countries of origin. However, the convergence of prevalence rates over time suggests that acculturation and the adoption of less healthy behaviors may be contributing to higher rates of CVD and angina among immigrants as they adjust to life in the US [18,19]. The observed fluctuations may reflect differences in healthcare access, lifestyle factors, or demographic shifts among foreign-born adults over time. Further, it is worth noting that acculturation-associated lifestyle changes, including increased consumption of processed foods and reduction in physical activity, along with socioeconomic stresses and limited culturally sensitive care, have been acknowledged to elevate the risk of cardiovascular conditions, including angina, among immigrants over time [18,19].

Geographic and socioeconomic disparities were particularly notable when analyzed through the CDC SVI and measures of income and education. Individuals in areas with high social vulnerability consistently showed the highest prevalence of angina, ranging from 1.5% to 2.1%, compared to those in areas with little to no social vulnerability, whose prevalence ranged from 1.3% to 1.7%. Similarly, individuals with lower educational attainment, such as those without a high school diploma, experienced the highest prevalence of angina (2.3% to 3.4%), while those with a college degree or higher consistently reported the lowest prevalence (1.0% to 1.2%). These patterns highlight the compounding effects of economic instability, limited access to healthcare, and health literacy on cardiovascular health outcomes [20,21]. Thus, the observed trends indicate that individuals with higher social vulnerability consistently experience a greater burden of angina pectoris, emphasizing the importance of targeted health interventions. Comparisons with nations such as Sweden and Canada have highlighted how universal healthcare and sturdy social support systems might result in divergent angina prevalence patterns and a reduction in health disparities [20,21].

Employment status was another important factor influencing angina prevalence. Individuals who were not currently employed but had worked previously showed the highest prevalence of angina, ranging from 2.9% to 3.6%. Conversely, employed individuals, particularly those working full-time, exhibited the lowest prevalence, consistently below 1%. These findings underscore the potential protective effects of stable employment, which may provide access to healthcare benefits, reduce stress, and encourage healthier lifestyle behaviors. However, part-time employees also demonstrated slightly elevated prevalence rates compared to full-time workers, possibly reflecting economic insecurity or limited access to comprehensive healthcare coverage [22].

Income level significantly influenced the prevalence of angina, with individuals living below 100% of the FPL experiencing the highest prevalence, ranging from 2.4% to 3.1%. In contrast, those with incomes at or above 200% of the FPL consistently exhibited the lowest prevalence, ranging from 1.1% to 1.4%. These findings highlight the role of economic disparities in shaping health outcomes, as lower-income individuals are more likely to face barriers to healthcare access, experience chronic stress, and have limited opportunities for preventive care and healthy lifestyle choices [6].

Lastly, comorbidities and health-related behaviors were strongly associated with angina prevalence. Individuals with hypertension, diabetes, or other chronic conditions were more likely to report angina compared to their healthier counterparts. Similarly, smoking and physical inactivity were significant contributors to angina prevalence. These findings reinforce the importance of addressing modifiable risk factors through targeted public health interventions and clinical strategies aimed at improving cardiovascular health across all population subgroups [23,24]. Healthcare quality and access might additionally confound socioeconomic and racial disparities in angina prevalence, given that underserved populations always experience diagnostic delays, misdiagnosis as a result of systematic barriers, and undertreatment [24].

Strengths and limitations

This study leverages a robust dataset from NHIS, providing comprehensive insights into the prevalence and trends of angina pectoris. The large sample size and consistent methodology ensure reliable and generalizable findings across diverse populations. By examining demographic, socioeconomic, and health-related factors, the study highlights key disparities, offering valuable guidance for targeted interventions. Moreover, the inclusion of multiple years allows for trend analysis, capturing evolving patterns and potential impacts of public health initiatives or socioeconomic shifts.

However, the study has limitations. The reliance on self-reported data introduces the potential for recall bias and underreporting, particularly for conditions with mild or unrecognized symptoms. The cross-sectional design limits causal inferences, and the absence of clinical validation of diagnoses may affect accuracy. Additionally, the study lacks granular data on regional and behavioral factors, which could provide a deeper context for the observed disparities. Also, there are limitations linked to the use of secondary data, including potential biases resulting from the self-reported measures that can introduce recall and reporting errors. Moreover, there was limited control with regard to data collection methods and variable definitions, which might affect consistency and comparability across the study years. Our analysis remained descriptive because it was cross-sectional, so we did not include multiple health system confounders from NHIS, such as insurance status or residence type, and self-reported healthcare measurements, since these could affect results. Future studies need to include these variables in multivariable or stratified analyses to validate the reported findings.

Further, it is worth noting that NHIS excluded institutionalized patient populations, including individuals admitted to nursing homes and long-term care facilities, which might lead to the underestimation of the prevalence of angina in older adults, who are a group at high risk for cardiovascular conditions. Other than the recall bias and underreporting, there is also a potential for selection bias, owing to the sampling frame, as well as misclassification bias, owing to increased dependence on self-reported diagnoses, which might not be reflective of the clinical assessments. The cross-sectional design also presents limitations, given that, even as it enables the observation of correlations at a single point in time, it is unable to aptly establish causal associations and effectively capture the temporal dynamics, including changes in health behaviors over time and cohort effects. There are also likely to be limitations in generalizability, especially for underrepresented regions and subpopulations that might be inadequately captured by the NHIS sampling, including non-English-speaking and rural communities. Lastly, there is a potential for unmeasured confounding variables, including chronic comorbidities, genetic predispositions, and psychosocial stressors, capable of influencing the observed correlations. As such, future studies should incorporate additional variables that include dietary habits, healthcare access, utilization patterns, and physical activity levels known to affect cardiovascular health and might refine the interpretation of the prevalence of angina and its various risk factors.

## Conclusions

The prevalence of angina pectoris is influenced by demographic, socioeconomic, and health-related factors, with higher rates among older adults, males, and those with lower socioeconomic status. Expanding healthcare access, especially for underserved communities, is crucial in reducing the burden of CVD. Prevention efforts should prioritize early screening, lifestyle modifications, and education to improve risk factor awareness and management. Addressing systemic barriers such as income inequality, employment instability, and healthcare disparities can help mitigate these trends. Community-based interventions and policy-driven approaches are essential for equitable healthcare outcomes. Strengthening these efforts will reduce health inequities, improve cardiovascular health, and enhance well-being. To build on the findings of this study, future research should explore emerging technologies and personalized medicine for early detection and management of angina. Moreover, future studies should strive to evaluate the social and biological determinants of angina disparities, focusing on healthcare access, genetic, and environmental factors. Additional longitudinal studies are needed to clarify the socioeconomic influences, even as randomized controlled trials are required to evaluate the various targeted interventions, including policy changes and community health programs. Additionally, to improve access to healthcare, policy interventions that include expansion of Medicaid coverage and integration of culturally customized health education programs, especially in groups considered high-risk such as low-income and ethnic and racial minority groups with reported higher CVD burdens. Furthermore, community-based contexts, including faith-based organizations and federally qualified health centers, play an active role as strategic locations for delivering targeted interventions. Lastly, to reinforce the existing evidence base, we recommend conducting additional cohort analyses and longitudinal studies to assess the long-term trajectories of risk factors and causal pathways. Such studies will aid in refining intervention strategies and inform future policy recommendations aimed at reducing cardiovascular health disparities.
